# Nutrition in New Zealand: Can the Past Offer Lessons for the Present and Guidance for the Future?

**DOI:** 10.3390/nu12113433

**Published:** 2020-11-09

**Authors:** Jane Coad, Kevin Pedley

**Affiliations:** 1 School of Food & Advanced Technology, College of Sciences, Massey University, Palmerston North 4442, New Zealand; 2 School of Health Sciences, College of Health, Massey University, Palmerston North 4442, New Zealand; k.c.pedley@massey.ac.nz

**Keywords:** nutrition science, food security, food systems, food policy, food industry, food additives

## Abstract

Over the last century, nutrition research and public health in New Zealand have been inspired by Dr Muriel Bell, the first and only state nutritionist. Some of her nutritional concerns remain pertinent today. However, the nutritional landscape is transforming with extraordinary changes in the production and consumption of food, increasing demand for sustainable and healthy food to meet the requirements of the growing global population and unprecedented increases in the prevalence of both malnutrition and noncommunicable diseases. New Zealand’s economy is heavily dependent on agrifoods, but there is a need to integrate interactions between nutrition and food-related disciplines to promote national food and nutrition security and to enhance health and well-being. The lack of integration between food product development and health is evident in the lack of investigation into possible pathological effects of food additives. A national coherent food strategy would ensure all components of the food system are optimised and that strategies to address the global syndemic of malnutrition and climate change are prioritised. A state nutritionist or independent national nutrition advocacy organisation would provide the channel to communicate nutrition science and compete with social media, lead education priorities and policy development, engage with the food industry, facilitate collaboration between the extraordinary range of disciplines associated with food production and optimal health and lead development of a national food strategy.

## 1. Introduction

Most years, a New Zealander, who has made a contribution to nutrition or related sciences in New Zealand, is invited to deliver the Muriel Bell memorial lecture [1] at the annual scientific meeting of the Nutrition Society of New Zealand, on a topic of their choice. The public lecture commemorates the life and work of Muriel Bell, 1898–1974. It is, almost always, easy to link the lecturer’s background, research interests and lecture content with Muriel Bell’s own interests and history, comprehensively described in a recent biography [2]. This might suggest that Muriel Bell worked in an extraordinary breadth of areas related to nutrition or that the same nutritional problems exist today—or both. This commentary aims to summarise the role of Muriel Bell in the history of nutrition in New Zealand, to consider how the nutrition landscape has changed since her era and to provide an overview of the nutritional challenges ahead and to consider how Muriel Bell might have responded.

## 2. Muriel Bell and the Early History of Nutrition in New Zealand

Whilst Bell’s history is known well by nutritionists within New Zealand, her work is less well known elsewhere. Bell qualified in 1922 as one of the first female doctors in New Zealand. The 1920s can be regarded as the birth of modern nutrition science. Following Funk’s postulation of the existence of “vital amines” in 1910 [3], diseases that had been thought to be due to infection or deprivation were found to be the result of vitamin deficiency. Between 1928 and 1943, five Nobel prizes were awarded in Physiology and Medicine for the discovery of vitamins and the elucidation of their mechanisms in preventing deficiency diseases. At Otago University, Bell carried out research in physiology and biochemistry, almost all of which was focussed on nutrition. The shortage of male medical graduates during and after the first world war created unprecedented opportunities for young women to become involved in university life and undertake teaching duties [2]. Bell’s medical dissertation was on basal metabolism and thyroid disease which was a significant problem. New Zealand soils have low levels of minerals, such as iodine, cobalt, fluoride and selenium, exacerbated by leaching from high rainfall, a situation that affects human and animal health.

After spending a few years working in Europe, Bell returned to New Zealand in 1935 where she was appointed to the Board of Health, with responsibility for the health of women and children, and to the New Zealand Medical Research Council. Bell became director of the Nutrition Research Unit at Otago University and took on the role of New Zealand’s first (and only) state nutritionist in 1940, a position she held until she retired in 1964. She advised the government about the post-war nutrition problems and food shortages and the setting of food rations and was instrumental in tackling problems of iron, calcium and iodine deficiency and was an avid campaigner for significant public health initiatives such as introducing the school milk scheme, promoting iodised salt and fluoridation of municipal water.

Bell was an inspiring communicator and used a broad range of strategies to convey her messages. For many years, she wrote a column about nutrition in a popular New Zealand magazine and gave numerous radio broadcasts. In two of her books, Bell identified nutritional issues which are still challenges today, 60 or so years after they were first published. The slim volume “Nutrition in New Zealand” [4] reviewed nutrition in New Zealand and identified three important factors “which determine the nutritional well-being of a country’s inhabitants”—whether there was enough food of the right kind containing the necessary nutrients, whether New Zealanders could procure this food and whether they knew how to choose and prepare the foods suitably. A subsequent and frequently revised book “Normal Nutrition” [5] described problems of the NZ “average diet” in 1969 and noted that the “average” New Zealander tends to eat too much sugar, cakes, biscuits and confectionery, too much butter, fat and cream, and that some people eat more meat than is necessary and some still do not use enough iodized salt. It concluded that the average New Zealander consumes too little milk, cheese, eggs, raw fruit and vegetables. Little has changed.

The history of nutrition over the last 100 years or so since the recognition of vitamins in deficiency diseases has been elegantly reviewed [6,7] and four phases identified. Muriel Bell witnessed phase 1, the discovery of vitamins and elucidation of their roles in deficiency diseases, and phase 2, the food shortages related to the economic depression of the 1930s and subsequent world war, resulting in the derivation of dietary requirements and drives to increase the production of food, to feed the increasing global population and the increasing risk of famines. The Green Revolution drove an extraordinary increase in food production, exceeding the growth in populations, so that many parts of the world overcame chronic food shortages and did not experience the predicted famines and malnutrition.

By the 1980s, nutrition in phase 3 was dominated by concerns about the increasing prevalence of chronic diseases, many associated with a dissociation between energy intake and energy expenditure which had never existed previously. Phase 3 of the history of nutrition, which was characterised by taking the same reductionist approach that had worked so well when determining the role of a single nutrient in a single deficiency disease, was not successful in elucidating a single and manageable factor affecting the increasing risks for non-communicable diseases such as cardiovascular disease and type-2 diabetes, which are affected by a combination of factors including dietary imbalance and energy excess. The current era (phase 4) is distinguished by an understanding that nutrition cannot be considered in isolation and that food systems need to be viewed holistically in the context of environmental sustainability and the broader costs of food production to optimize personal, public and planetary health [8].

## 3. Nutritional Challenges Today (Phase 4)

The nutritional challenges of the current era are complex. The global burden of disease has shifted from infectious diseases and nutrient deficiency disorders to noncommunicable diseases (NCDs) which are the main health and development challenges in the world. The recognition of the coexistence of undernutrition (stunting and wasting, often compounded by micronutrient deficiency) with overnutrition (overweight and obesity associated with increased risk of diet-related noncommunicable disease) led to the concept of the “double burden” of malnutrition [9]. It is appreciated that the greatest threats to human health and survival, obesity, undernutrition and climate change are components of a global syndemic that affects most people in the world [10]. The challenge of reducing the double burden of nutrition and securing a healthy sustainable future for the growing number of the world’s inhabitants is paramount.

The globalisation of the environmentally costly food system is associated with the production of inexpensive and low-quality foods, which are often energy-dense and nutrient-poor. Feeding the future global population requires eating habits to be transformed, food waste to be reduced and food production to be improved, at least partially, by adopting creative and locally rational farming methods and unlocking the genetic potential of neglected and underutilised species which may be more resilient to climate change. Climate change is predicted to affect the nutrient composition of crops; 85% of plants have C3 metabolism (utilise the 3-phosphoglycerate pathway in photosynthesis) and so have lower levels of protein, iron and zinc when carbon dioxide levels are higher [11]. About 90% of the world’s food comes from less than 1% of the known 250,000 edible plant species [12], mostly (>75%) from 12 species of plants and 5 species of animals [13].

To date, the production of food has apparently kept up with population growth, but in 2019, prior to the negative effects that COVID-19 is likely to generate, more than 690 million people (8.9% of the global population) were undernourished [14]. Furthermore, 2 billion people (25.9% of the global population) experienced hunger related to food insecurity and a lack of reliable access to nutritious food, and a further 2 billion people were overweight or obese [15]. These numbers have been slowly increasing and are projected to continue to do so, at least until 2030. Despite the 1943 UN Conference on Food and Agriculture setting a goal of “freedom from want of food…. within the shortest possible time” [16], the same issues were prevalent in 2000 when the Millennium Development Goal 1 (eradicate extreme poverty and hunger by 2015) and in 2016 when Sustainable Development Goal 2.1 (“to end all forms of hunger and malnutrition by 2030”) were established as targets; child malnutrition (wasting, overweight and micronutrient deficiency) and adult obesity continue to increase. Malnutrition is responsible for 41% of all deaths—10.9 million from chronic disease and 3.2 million due to maternal and child undernutrition [7]. It is unlikely that malnutrition will end by 2030 [17]. Whilst it is estimated that enough food is currently produced to feed the population of the world, over 1.5 billion people cannot afford the cost of adequate essential nutrients, and 3 billion cannot afford the cheapest healthy food. Furthermore, mortality due to NCDs is now higher than deaths due to communicable diseases, poor nutrition and maternal and perinatal conditions combined [18].

**Figure 1:**
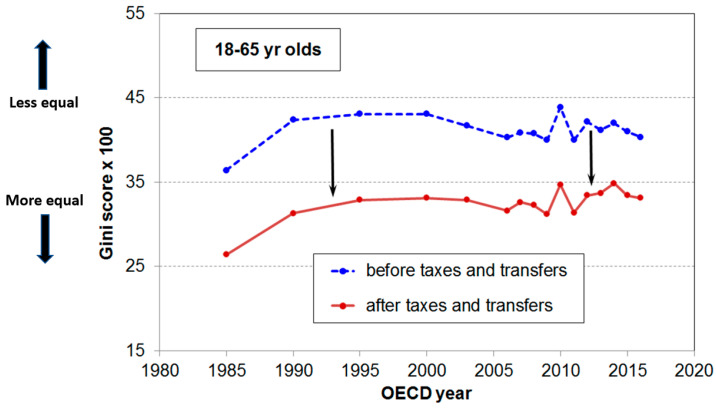
Gini score (x100) for total income before and after taxes and transfers in New Zealand. Between 1960–1990, there was little change in inequality followed by a steep rise which was associated with income tax and benefit changes. Reproduced with permission from [19]. Copyright Ministry of Social Development, New Zealand, 2019.

Increased food production does not, however, mean less hunger and better nutrition; poverty, not the physical shortage of food, is the primary cause of hunger. In Muriel Bell’s time, NZ was a more equitable society. In the 1960s, New Zealand had one of the highest income per capita in the world, and it was evenly distributed [4]. This situation changed dramatically over subsequent decades. Income inequality in New Zealand, described by the Gini coefficient for total incomes of all individuals before tax, was relatively stable from the early 1960s until the mid 1980s, after which it increased steeply (Figure 1).

Like many other countries in the world, the ”wealth gap”’ between the richest and poorest in New Zealand society continues to escalate with recent reports suggesting that the richest 1% are worth 68 times more than a typical New Zealander and the wealthiest 10% own nearly 60% of all assets [20].

**Figure 2:**
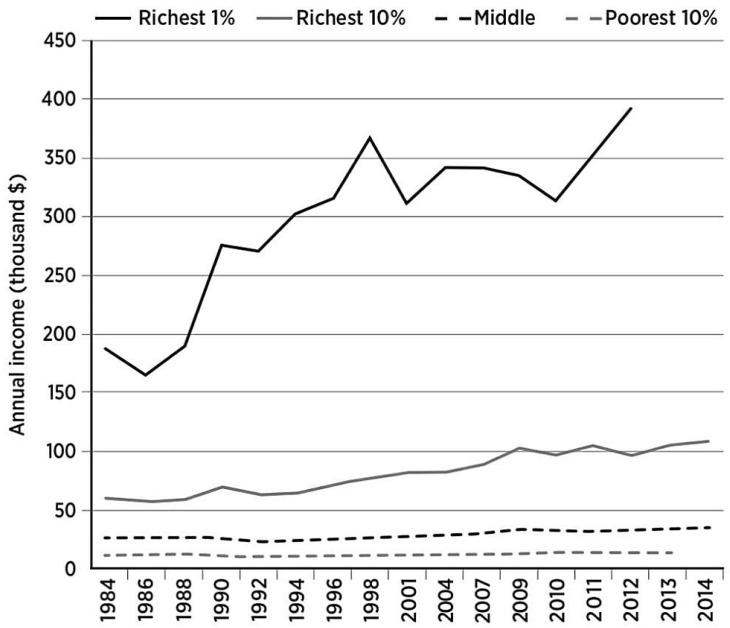
Annual average disposable incomes for 4 groups in New Zealand shown in 2012 NZD equivalents and adjusted for inflation rates. Steep rises for the richest 1% contrast with very small increases for the poorest 10%. Reproduced with permission from [23]. Copyright Bridget William Books, Wellington, New Zealand, 2015.

**Figure 3:**
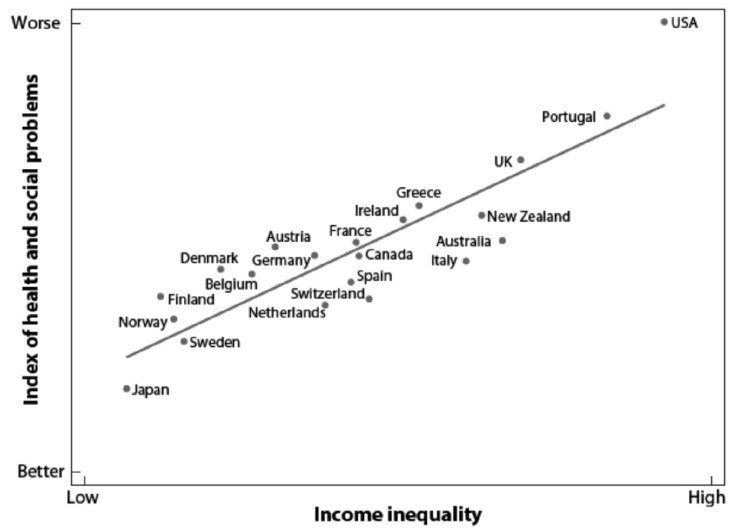
Relationship between income inequality versus health and social problems in wealthy countries. Income inequality is measured as the ratio of incomes between the richest 20% and poorest 20% in each country. Data are based on life expectancy, mental illness, obesity, infant mortality, teenage births, homicides, imprisonment, educational attainment, distrust and social mobility. Raw scores for each variable were converted to z-scores and each country given its average z-score. Reproduced with permission from [23]. Copyright Penguin Books, 2009.

Based on the Gini data from the last 25 years, inequality in New Zealand appears to have remained fairly constant; however, the wealth of the country has increased markedly and most of the benefits of that wealth have been to those on higher incomes (Figure 2). Not surprisingly, the diversity between the ”haves” and the ”have nots” has a major impact on diet and, therefore, on health and wellbeing. The notion that New Zealand is egalitarian is simplistic. There are many health issues (Figure 3) which are clearly associated with income inequity [21]. In developed countries, income inequity has been reported to have an association with obesity, mental illness, life expectancy and infant mortality as well as a range of social issues [22].

A major consequence of income inequality is its negative impact on food security, the ready access to adequate food supplies, which is a major and growing concern worldwide. It is believed that sufficient food is currently produced in the world to feed the population [24]. The case for increasing global food production to feed a continuously growing world population may be encouraged by the food and agricultural industries whose claims are not independent of profit.

## 4. Population Expansion and Increased Requirement for Food

United Nations data predict that the population of the world will increase from 7.7 billion in 2019 to almost 8.5 billion by 2030, 9.7 billion by 2050 and 10.9 billion by 2100 [25]. Improved health and nutrition together with significant reductions in the mortality of children resulted in an extraordinary growth of the world population from the beginning of the 20th century. However, the rate of global population growth is declining after peaking to over 2.1% in 1968; rate of growth is abrogated by access to contraception and falling fertility [26] together with the increased schooling of children, particularly girls. In the past, the size of the population was limited by very high mortality rates; in the future, it will be restrained by low fertility. The modelling indicates that the global population will have increased more than 10-fold over 250 years with the projected world peak numbers being reached at the beginning of the 22nd century.

The massive demographic transition of reaching about 10 billion people by 2050 significantly increases the challenge of matching population growth and demand for food. It is modelled that global food demand will require a substantial 60–70% increase in food production [27] with corresponding demands placed upon agriculture and horticulture, land availability and soil fertility, and a food industry capable of providing the food requirements of the expanding and largely impecunious population. While it is evident that there will be more people to feed, there is no consensus about how substantial the increase in food production will need to be. The most significant increases in population growth, accompanied by a shift from rural to urban living, will occur in countries which are currently poor and have low per capita food consumption. Understanding of nutrition transition, how diets change as income increases, indicates that there will be an increase in noncommunicable diseases that results from the changes classically described by Engel’s Law (that the proportion of income spent on food diminishes as income increases) and Bennett’s Law (that nutrient density increases—or the starchy-staple ratio falls—as income increases) [28]. Traditionally, this has been interpreted as the demand for animal-based foods will increase to a greater extent than the increase in population growth. However, the drive for societal adaptation [29] to reduce the consumption of animal-based foods would further increase the availability of human-edible crops, as would making dietary changes such as adopting one of the variants of the EAT-Lancet Commission reference diet [30].

Furthermore, reductions in food waste would also have a positive impact on the food balance. About a third of all food currently produced in the world (1.3 billion tonnes per year) is wasted [31]. Most wasted food ends up in landfill and contributes to greenhouse gas emission. For example, New Zealand households discard 157,389 tonnes of food waste each year (worth about NZD 1.17 billion/year, USD 0.77 billion/year) to kerbside rubbish collection [32]. This is just a proportion of total food wasted as there has not yet been a nationwide audit investigating all stages of the food supply chain. The waste is classified as avoidable (could have been eaten) like bread, potentially avoidable like potato skins or unavoidable like banana skins. It is not known how much household food is composted, fed to animals or disposed of into the wastewater system. These figures do not include supermarket food waste, some of which may be sent to food rescue groups or used for animal feed, or waste from the hospitality sector. As in many countries, New Zealanders buy too much food, store it poorly, cook too much and usually do not eat leftovers.

## 5. Environmental Costs of the Food Systems

Meeting human food requirements while maintaining healthy ecosystems is one of the greatest challenges of the 21st century. In the 10,000 years between the birth of agriculture and 1960, food grain production reached 1 billion tonnes [33]; only 40 years later, it had doubled to 2 billion tonnes. The post-war concerns about the world’s ability to feed itself and the dire predictions of large-scale famines were addressed by the Green Revolution. The dramatic increase in food production was achieved by the development of new technologies such as breeding of high-yielding cultivars of wheat, rice and maize which were dependent on a high input of agrochemicals, fertilisers, pesticides, irrigation systems, mechanical cultivation techniques and fossil fuels.

The EAT-Lancet Commission report [30] drew attention to both the importance of nutrition in optimizing human health and the impact of global food systems on planetary health and called for substantial dietary shifts in order to facilitate the transformation to sustainable food systems delivering healthy diets. A staggering 25% of greenhouse gas emissions, 32% of energy used, 69% of fresh water used, 80% of deforestation and compromised quality of the soil and oceans are the cost of our current food production [7]. The loss of biodiversity negatively influences food security, clean water and raw materials; the cost is borne primarily by the poor and most vulnerable. The way food is produced is emerging as a major threat to development and health [34]; the global food system has crossed several of the “planetary boundaries” (rate of biodiversity loss, amount of nitrogen removed from the atmosphere and climate change) identified as being essential for life on earth to be sustainable [35]. Mono-cropping has resulted in genetic erosion and depletion of soil nutrients.

Over 95% of the food grown in the world is grown in topsoil which is fast disappearing due to soil erosion and degradation; in the USA, soil erosion is estimated to be about 8-fold higher than the rate of soil formation [36]. The rapid loss of topsoil has been suggested to imperil both the quantity and nutrient quality of crops in coming years as well as water quality and carbon retention. Several contributing factors to this loss include removal of vegetation, intensive tilling which removes organic matter and nutrients, wind, drought and the increased and regular use of fertilisers and pesticides. Land degradation and unsustainable soil management practices, including intensive use of synthetic nitrogen fertilisers, have led to the world’s cultivated soils losing 25–75% of their original carbon content; this is lost into the atmosphere and amplifies climate change [37]. Sustainable soil management approaches focus on maintaining carbon rich soils and promoting sequestration of carbon which help mitigate greenhouse gas emissions and food insecurity thus benefitting food production.

Over recent decades, there have been dramatic advances in food handling, preparation and storage, use of insecticides and surveillance of food-related diseases. However, there are new threats that affect farm animals which used to be either rare or restricted such as the outbreaks of bovine spongiform encephalopathy (BSE or mad cow disease) in the 1980s and foot and mouth disease in the 1990s in the UK. The use of antibiotics as growth promotors and in food packaging has seen unprecedented increases in antibiotic resistance. Changes in balance between human food acquisition as a result of decreased hunting and increased farming, the shift to urban living and decreased biodiversity has increased human contact with wildlife and increased the risk of zoonotic disease; it is estimated that over 60% of known infectious diseases and 75% of new infectious diseases come from animals [38]. In addition, there is increasing awareness that foods (and water) are vulnerable to malevolent or deliberate tampering and bioterrorism. Although there have been significant advances in food preservation and packaging, it is evident that food safety issues have the potential to affect trade and are, therefore, subject to political influence. Government actions are usually in favour of the interests of industry, rather than protecting the consumer or public health interests [39].

## 6. The Influence of the Food Industry

The 2012 PLoS Medicine series about Big Food started with the candid observation that “Global food systems are not meeting the world’s dietary needs” [40]. Food companies are businesses, not public health agencies set up to deliver optimal human nutrition; their allegiance is to their shareholders and their foremost aim is to maximise their profits. This apparent conflict of interests is likely to increase, not decrease, with population growth leading to even greater health disparities.

Modern food systems are ruled by “Big Food”, the huge and powerful transnational food, beverage and supplement companies, which has the primary objective of maximising sales and profits. About 75% of world food sales involve processed foods; ultraprocessed foods account for more than half of the energy intake in high income countries [41]. The foods people have access to and eat are increasingly controlled by surprisingly few transnational corporations. The growth in this market is predominantly in low- and middle-income countries and the food sold has fuelled nutrition transition and displacement of traditional long-established food systems and dietary patterns to highly processed foods which compromise the nutritional quality of the diet and have low satiety potential, high glycaemic responses and promote dysbiosis of the gut microbiota. There is increasing evidence that the powerful and well-resourced food and beverage companies use similar strategies as Big Tobacco to undermine/weaken public health initiatives such as taxation of sugar-sweetened beverages and regulation such as protecting children from targeted advertising [42]. The food industry funds not-for profit community-based opposition groups, funds academic research to support claims, sponsors events for scientific and health organisations, and shapes consumer preferences through aggressive marketing practices. The research studies funded by food companies invariably publish results that benefit the funder and often influence dietary advice [43].

Many forms of food processing are beneficial to health but ultraprocessing creates highly palatable, cheap, ready to eat products that are characteristically energy dense and high in refined starches, sugars, fats, additives and salt, as well as being highly profitable and highly promoted. These products dominate the food supplies of high-income countries, and their consumption in low- and middle-income countries is increasing dramatically and contributing to the patterns of nutritional transition. The tension with which obesity is viewed as a personal responsibility (poor self-control, laziness and personal failing) and the food companies’ emphasis on choice and individual agency, leaving government agencies to promote diet and exercise, results in neutralisation of inconvenient legislation and policies and ignores the impact of the obesogenic environment. It creates a framework of legitimacy for the promotion and consumption of ultraprocessed foods and suggests that with an active lifestyle the food or food industry is part of the solution. The modern response of many food companies (described as Corporate Social Responsibility) to criticism is to engage in activities which encourage people to take up a healthy and active lifestyle. This promotion of fitness presents a marketing platform which is hard to challenge. For instance, the Global Energy Balance Network, sponsored by one of the world’s largest providers of sugar-sweetened beverages, was established as a non-profit organisation purporting to fund research into obesity but insidiously promoting the notion that lack of exercise, not diet, was the main cause of obesity [44]. Public–private partnerships between the food industry and nutritionists imply a shared health goal and suggest a claim that the food is part of the solution to the problem, but this can be a nutrient-based minor modification or reformulation to a product which makes it less bad but creates a health halo.

New Zealand is a major global food and beverage exporter capitalising on exports [45]; the food and beverage industry has a total revenue of NZD 71.7 billion which accounts for 46% of all goods and services exported. Food supply is driven by profit and sometimes by competition between export and national consumption. New Zealand is one of the largest dairy producers in the world, exporting much of its produce overseas. Whereas dairy consumption (milk and yoghurt) accounts for approximately 20% of food costs for a family of four in New Zealand [46], milk in New Zealand is approximately twice as expensive as milk purchased in central London, despite New Zealand’s 20-fold higher production than the UK, per capita.

There is an urgent requirement for nutrition and food technology to be more integrated. Without the input of technological approaches to develop new foods such as those based on novel protein sources such as insects and fungi, it is unlikely that the target of feeding the world population can be met. However, food technologists need to appreciate nutritional physiology and the potential damage to health that might result from the increasing use of food additives that have negative and under-appreciated effects on human health. Recent years have witnessed a major interest in the cellular mechanisms that are associated with the links between dietary intake and a range of human diseases. These links comprise direct effects of nutrient and non-nutrient additives on human cells and, indirectly, on the enormously important microbiome, the composition of which is intimately linked to human health. The microbiota exceed the number of human cells present in the body by approximately 10-fold and play a vital role in several body tissues with a major impact in the human gut.

Understanding the causes and effects of dietary components such as ultraprocessed foods, emulsifiers and non-nutritive (or artificial) sweeteners (NNS) on human health requires an understanding of their direct effects on human cells, as well as on the composition and function of the commensal microflora. Changes in this composition can have an enormous impact, for example, on gut barrier function, and is implicated in a range of human disorders including Crohn’s disease and coeliac disease. An understanding of the interactions between the negative effects of NNS on gut barrier function, gut microbiota composition and other potential pathological effects of these dietary supplements may shed light on their possible involvement in inflammatory bowel disease (IBD) and other gut disorders [47]. Thorough testing of food additives before their approval for use by the food industry should always examine their possible effects on the gut microbiome, a crucial assessment which is very seldom done.

Non-nutritive sweeteners have detrimental effects on metabolism because they increase caloric sugar absorption, interfere with gut microbiota, induce glucose intolerance, disrupt tight junction integrity and gut barrier function and interact with sweet-taste receptors expressed throughout the gut that play a role in glucose absorption and insulin secretion, thus interfering with learned responses that contribute to control of glucose and energy homeostasis. NNS are metabolised in the gut; there are concerns that the metabolites produced may be mutagenic or toxic at high concentrations.

Coeliac disease (CD) is a multifactorial autoimmune disorder in which the ingestion of gluten, in subjects with a genetic predisposition, triggers the movement of gluten peptides through the gut barrier where they evoke an immune response. The incidence of CD is increasing in some countries; this may be influenced by an increased awareness of symptoms and improved diagnosis, but may also result from the involvement of environmental factors other than gluten. The movement of gluten peptides across the epithelial barrier can result from increased transcellular transport of gluten or increased paracellular leakage of gluten through the normally impenetrable tight junctions. Any dietary components that increase these transport processes may be involved in enhancement of the autoimmune response and increased gut pathology. Similarly, dietary components that increase the subepithelial processing of gluten by transglutaminases, or the presentation of the deamidated gluten products to target immune cells, would be expected to enhance the autoimmune response. Microbial transglutaminases, used as food additives to increase water retention and improve food texture, can mimic the antigenic gluten products that trigger CD [48]. Other possible food additives which may be implicated in the increasing incidence of CD include compounds, such as metallic nanoparticles and emulsifiers, which undermine gut barrier integrity and allow otherwise impermeable gut contents to enter the subepithelial space and trigger an immune response [49].

Dietary emulsifiers, used to improve flavour, texture and stability of processed food, have deleterious effects on gut barrier function by increasing intestinal permeability via paracellular and/or transcellular routes, as well as by their mucolytic activity which breaks down the protective mucus layer which covers the gut epithelium. The commonly used emulsifier, polysorbate 80, was shown to increase *Escherichia coli* translocation across M-cell and Caco2 cell lines [50]. It is speculated that the adoption of more westernised diets containing emulsifiers in a number of countries, such as Japan, might underlie the increased incidence of Crohn’s disease seen over recent years [51]. More recent studies have reported that the commonly used emulsifiers, polysorbate 80 and carboxymethylcellulose, induced changes in the composition of gut microbiota which triggered both inflammation and metabolic syndrome in mice [52]. Accumulating evidence suggests that food additives may similarly contribute to the pathogenesis of inflammatory gut diseases in humans [53,54].

Crohn’s disease is a chronic, idiopathic, inflammatory bowel disease (IBD) of the gut for which there is no cure. Worldwide, the incidence of Crohn’s disease is increasing, and it varies markedly between countries, with Canterbury in New Zealand having one of the highest incidences in the world [55]. The underlying mechanisms of IBD include a genetic predisposition together with an unidentified environmental trigger. Several possible triggers have been suggested including compromised epithelial barrier function leading to increased intestinal permeability, which allows access of commensal bacteria to the submucosa and dysbiosis between harmful and protective bacterial populations [56]. Mycobacterium avium subspecies paratuberculosis (MAP) is the pathogen that causes Johne’s disease in ruminants, which has a strikingly similar pathology to Crohn’s disease [57]. MAP infection, which is common in New Zealand and estimated to cost New Zealand NZD 40-88 million in lost animal production each year, has been repeatedly suggested to be an environmental trigger of Crohn’s disease in humans. Accumulating evidence for the involvement of MAP would explain the high incidence of Crohn’s disease in NZ and suggest a more careful approach to milk refinement and consumption to address the issue that MAP has been repeatedly identified even in pasteurised milk [58], albeit at reduced levels compared with unpasteurised milk.

In recent years, the prevalence of obesity has increased dramatically. Infants, born to obese mothers of low socioeconomic status, who are not breastfed, are more likely to have poor diets and become overweight [7]. It was recently estimated that if breastfeeding could be increased to near-universal levels worldwide, more than 800,000 lives could be saved each year, mostly of children under 5 years of age, as well as more than 20,000 deaths prevented per annum from breast cancer in mothers [59]. Breastfeeding has been correlated with improved intelligence in adulthood [60] which may have positive effects upon adult prosperity and health; exclusively breastfed infants are 14 times less likely to die than babies who are not breastfed. Numerous studies underpin the need for early adoption and continuation of breastfeeding to optimise infant and adult health [61]. Human milk provides optimal nutrition for a neonate with immature digestive, hepatic and renal systems providing not only the essential macronutrients, vitamins and minerals but also non-nutrients including growth factors, hormones and immune factors. It contains over 900 milk proteins [62] which include immune factors such as secretory IgA that are vital to neonatal defence. Secretory IgAs reflect the immune responses that have been raised in the mother to infective challenges, usually in a similar external environment to that in which the infant is born and offer passive immunity by coating the mucosal surfaces of the gut and other mucosae of the immune-deficient neonate. Despite these well-established benefits of breastfeeding to both offspring and maternal health, the food industry in New Zealand and worldwide continues to persuade pregnant and postnatal mothers to avoid breast feeding and purchase their formula milk, despite an inevitable health risk to both mothers and their offspring.

## 7. The Need for Nutrition Education

Sixty years ago, Muriel Bell identified a lack of satisfactory knowledge of how to choose and prepare foods [4] as one of the main limiting factors of the nutritional well-being of New Zealand’s inhabitants in the 1960s. Today, it is not just a lack of information, it is also the era of misinformation or “fake news” in which the instantaneous communication via the internet can be amplified and sensationalised in a way that makes valid scientific information difficult to identify. Increased nutrition literacy and knowledge are essential to avoid the potential for dramatically deteriorating population health as the food demands of the growing population increase. Nutrition literacy, defined as an individual’s ability to attain, process and understand nutrition information and the skills required to make appropriate nutrition decisions [63], is a vital component of achieving a healthy diet. As well as awareness of nutritional principles, practical skills such as food label reading, assessing portion size and preparing food are essential for health. Every child has the right to good nutrition and a healthy eating environment that supports life-long food preparation skills and acquisition of knowledge-based nutritional competence.

Currently in New Zealand, tertiary qualifications in nutrition are offered from four universities. Predominantly the qualifications offered prepare future graduates for working in dietetics, public health and sports-related roles. There is an urgent need to take a holistic approach and to extend the breadth of nutrition in tertiary education which will allow students to combine the study of nutrition with disciplines which are fundamental to an integrated nationwide approach to improve the nutrition of the population, to consider the health implications of foods being developed for national consumption and export and the repercussions for sustainable production of food. Furthermore, tertiary education qualifications in any subject related to food such as agriculture, horticulture, soil science, engineering and food technology, product development, business studies, entrepreneurship or economics should include nutrition. A review of the number of graduates aware of nutrition and able to play a role in the many different and fast changing areas related to sustainable food production is important. New Zealand already has a low density of nutrition professionals: 14.1 per 100,000 of population which is markedly below that of the UK and USA and almost half of the number in Australia [64]. It is essential that both the tertiary education and continuing professional development offerings for the employees in the food- and health-related sectors include basic nutrition and continual updating.

## 8. Conclusions

New Zealand is a significant food producer, and many New Zealanders are employed in food-related industries, as well as in health-related occupations. A coherent and coordinated national food policy is required in New Zealand to ensure all components of food systems are optimised and that strategies to address the global syndemic of malnutrition and climate change are prioritised. Such an approach would integrate policies relating to agriculture, social programmes to reduce food insecurity and hunger, education, food regulation and safety, trade and science research.

Muriel Bell was the only state nutritionist ever and a formidable campaigner who styled herself as “Battle-Axe Bell” [2]. Today, more than ever, New Zealand needs a state advocate or advocacy structure for nutrition. The voice of nutrition in New Zealand is alarmingly quiet. There have been several noteworthy international nutrition initiatives over recent years; even the UN Decade for Action on Nutrition (2016–2025) began with almost no comment or consideration in New Zealand, other than a couple of press releases from universities. New Zealand needs an independent nutrition sector that the public can rely on to explain why nutrition conclusions and recommendations change and why this is a sign of a healthily evolving scientific discipline like any other branch of medicine. A nutritional advocacy organisation could provide the infrastructure and an authoritative voice to ensure nutrition was considered in everything that affects public and planetary health such as trade and policy investments, and to provide leadership to ensure integrity, insight and inspiration in the conduct of food and nutrition collaborations in the public, non-profit and private sectors. Nutrition has never faced greater challenges; nutritionists in New Zealand should be well-placed to address them.
